# Supplementary figures and images for: Multi-Omics Analyses Revealed GOLT1B as a Potential Prognostic Gene in Breast Cancer Probably Regulating the Immune Microenvironment
Source: Front Oncol. 2022 Jan 19;11:805273. doi: 10.3389/fonc.2021.805273 (PMC8815109; doi:10.3389/fonc.2021.805273)

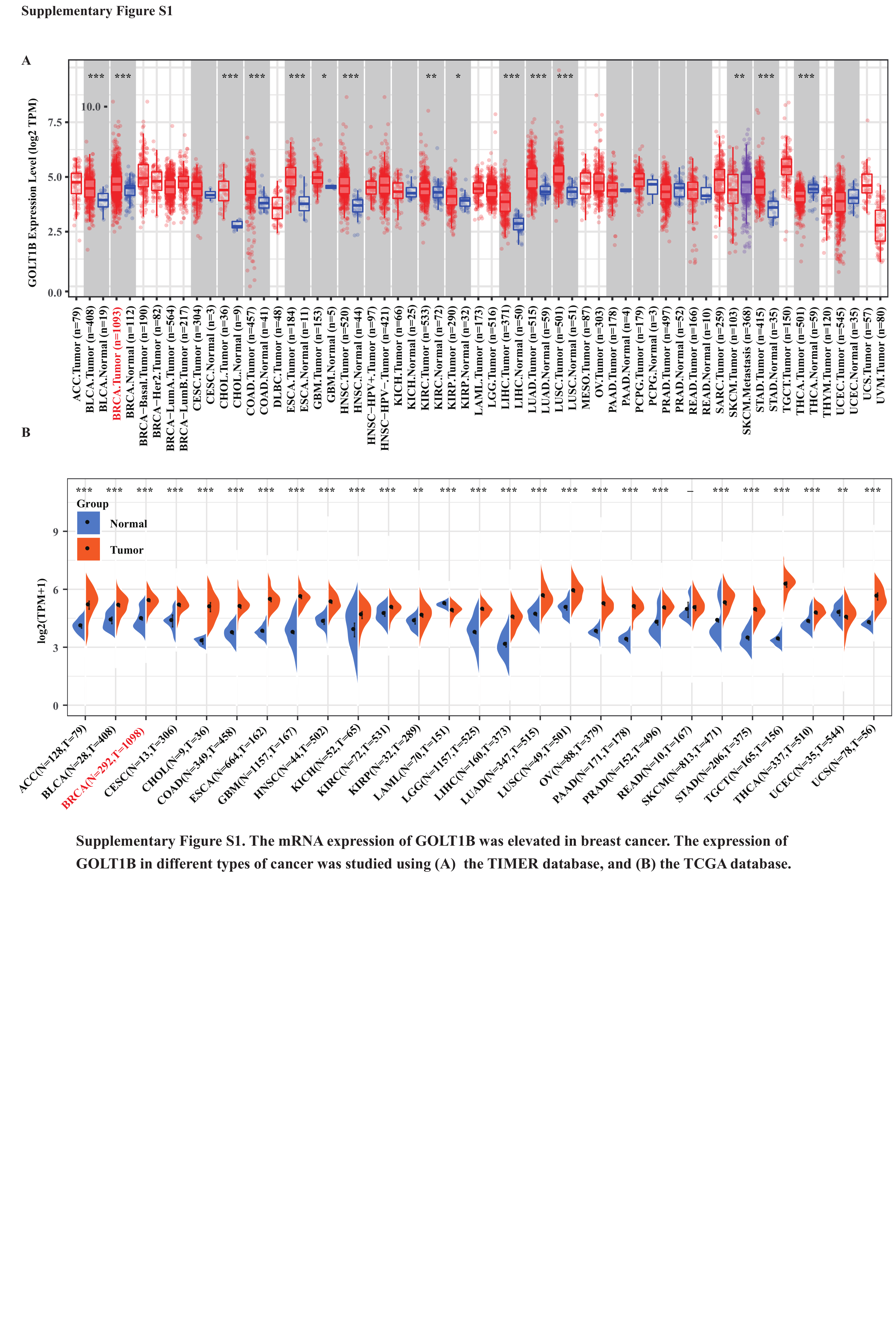

Supplement: Supplementary file 2 [file Image_1.tif]

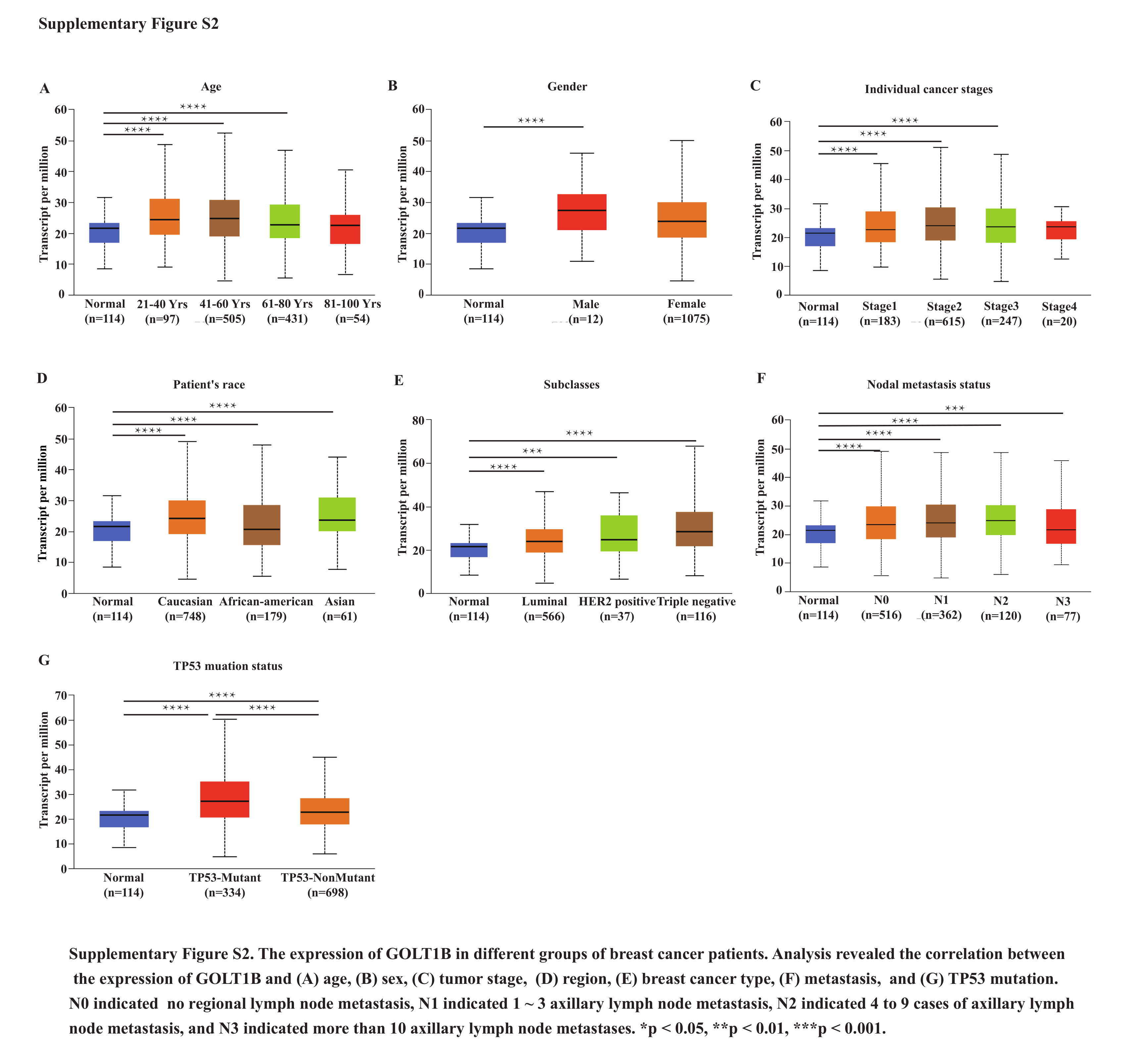

Supplement: Supplementary file 3 [file Image_2.tif]

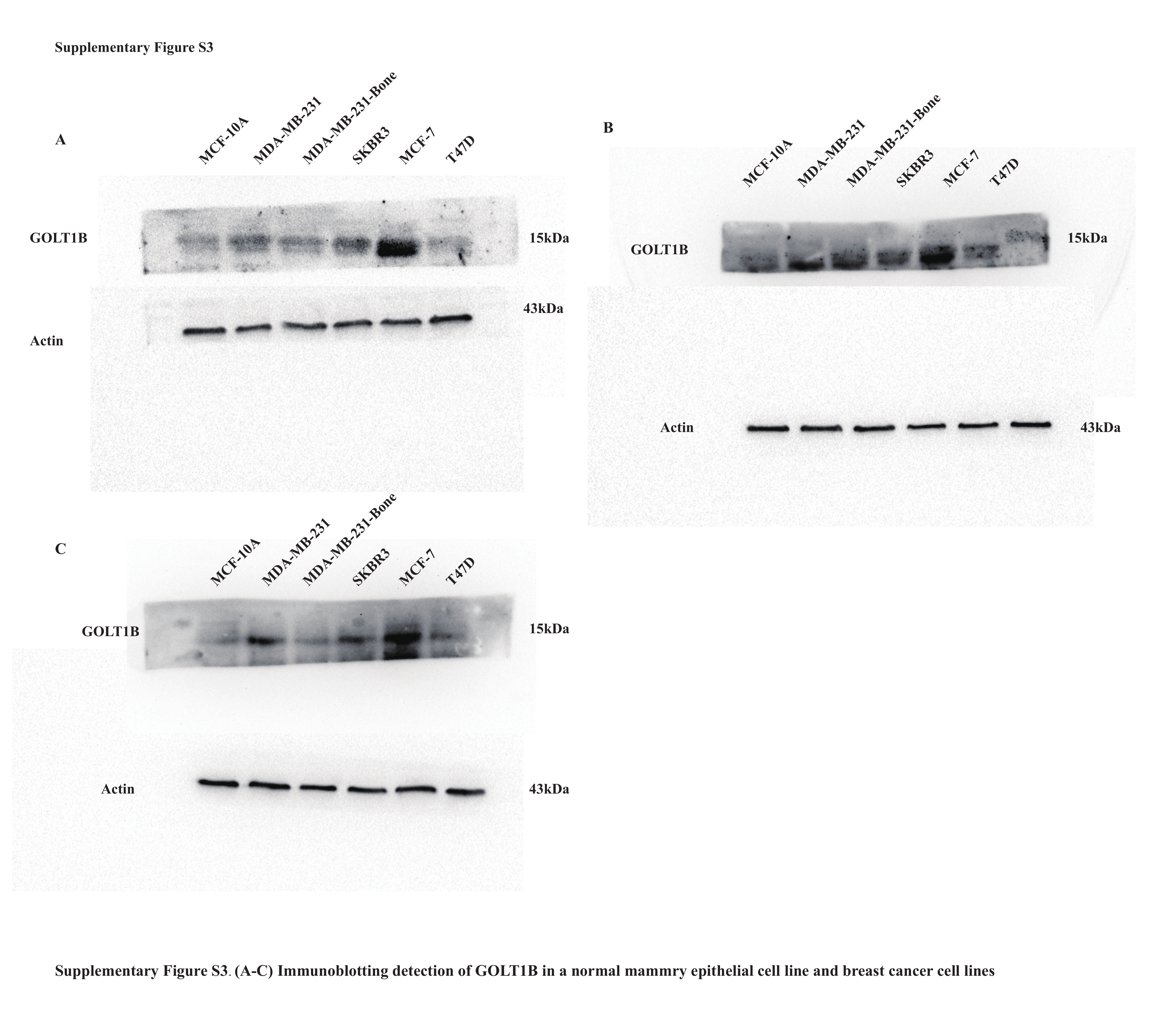

Supplement: Supplementary file 4 [file Image_3.tif]
